# Functional Involvement of Carbonic Anhydrase in the Lysosomal Response to Cadmium Exposure in *Mytilus galloprovincialis* Digestive Gland

**DOI:** 10.3389/fphys.2018.00319

**Published:** 2018-04-04

**Authors:** Roberto Caricato, M. Elena Giordano, Trifone Schettino, M. Giulia Lionetto

**Affiliations:** Department of Biological and Environmental Sciences and Technologies, University of Salento, Lecce, Italy

**Keywords:** carbonic anydrase, lysosomes, *Mytilus galloprovincialis*, cadmium, metals, mollusks

## Abstract

Carbonic anhydrase (CA) is a ubiquitous metalloenzyme, whose functions in animals span from respiration to pH homeostasis, electrolyte transport, calcification, and biosynthetic reactions. CA is sensitive to trace metals in a number of species. In mussels, a previous study demonstrated CA activity and protein expression to be enhanced in digestive gland by cadmium exposure. The aim of the present work was to investigate the functional meaning, if any, of this response. To this end the study addressed the possible involvement of CA in the lysosomal system response of digestive gland cells to metal exposure. The *in vivo* exposure to acetazolamide, specific CA inhibitor, significantly inhibited the acidification of the lysosomal compartment in the digestive gland cells charged with the acidotropic probe LysoSensor Green D-189, demonstrating *in vivo* the physiological contribution of CA to the acidification of the lysosomes. Under CdCl_2_ exposure, CA activity significantly increased in parallel to the increase of the fluorescence of LysoSensor Green charged cells, which is in turn indicative of proliferation and/or increase in size of lysosomes. Acetazolamide exposure was able to completely inhibit the cadmium induced Lysosensor fluorescence increase in digestive gland cells. In conclusion, our results demonstrated the functional role of CA in the lysosomal acidification of *Mytilus galloprovincialis* digestive gland and its involvement in the lysosomal activation following cadmium exposure. CA induction could physiologically respond to a prolonged increased requirement of H^+^ for supporting lysosomal acidification during lysosomal activation.

## Introduction

Carbonic anhydrase (CA) is a zinc metalloenzyme, which catalyzes the reversible hydration of CO_2_ in the following reaction: CO_2_ + H_2_O ⇌ H^+^ + ${\text{HCO}}_{3}^{-}$. It is widely distributed in nature from bacteria to humans with its 6 unrelated families (α, β, γ, δ, ζ, η) and its activity is required for a number of physiological processes (Lionetto et al., 2016; Supuran, 2016). In animals, it plays a key role in respiration, pH homeostasis, electrolyte transport, bone resorption, calcification, and biosynthetic reactions requiring bicarbonate.

In mammals, at least 16 different α-CA isoforms have been identified and several novel isoforms have been disclosed in lower vertebrates and invertebrates, although invertebrate isoforms are by far less investigated. In mollusks several α-carbonic anhydrases (α-CAs) isoenzymes have been described, which are involved in biomineralization, respiration, and pH homeostasis (Marie et al., 2008; Norizuki and Samata, 2008; Connor and Gracey, 2011; Dickinson et al., 2012; Le Roy et al., 2012; Ren et al., 2014). Most of the available information on CA in mollusks comes from gills (Cudennec et al., 2006; Pavičić-Hamer et al., 2016) and in particular from mantle, which expresses different isoforms involved in shell formation (Le Roy et al., 2012; Werner et al., 2013).

CA is known to be sensitive to trace metals, which represent one of the main classes of environmental pollutants. Trace metals inhibit CA activity in a number of tissues in a variety of animal species (Lionetto et al., 1998a, 2000, 2006, 2012a,b, 2016; Soyut et al., 2008; Kolayli et al., 2011; Kaya et al., 2013; Mercan et al., 2014) by displacement of Zn^2+^ in the active site or by binding to histidine and cysteine residues in other sites. For example, in the mantle of the filter-feeding mussel *Mytilus galloprovincialis*, CA was significantly inhibited by the exposure to 1.78 μM Cd^2+^ for 15 days (Lionetto et al., 2006). In the gills of the freshwater bivalve *Anodonta anatine*, CA activity was significantly decreased by the exposure to 0.35 μM Cu^2+^ for 2 weeks (Santini et al., 2011). However, the relationships between CA and trace metals are complex and multifaceted (Lionetto et al., 2016). In fact, the inhibition exerted by some metals on CA-specific isoforms does not explain all of the biological effects of metals on CA. In mussels, Caricato et al. (2010) demonstrated that cadmium exposure enhances CA protein expression and in turn CA activity in digestive gland (Caricato et al., 2010; Lionetto et al., 2014), although the functional role of this response, if any, remains still unknown.

The aim of the present work was to investigate the physiological significance of CA induction following *in vivo* cadmium exposure in mussel digestive gland, in order to increase the knowledge on the multiple functional roles played by CA in animal physiology.

It is recognized that mussel digestive gland accounts for the intracellular and extracellular digestion of nutritive substances, storage of glycogen, lipids, proteins (Bayne, 1976), and detoxification and accumulation of both essential and toxic metals. The digestive cells, the most abundant cell type in the digestive gland, are characterized by the presence of a well-developed lysosomal system, which functions as a digestive system. The mussel lysosomal system is known to react to pollutant exposure, including metals, through alteration in lysosomal fusion events, changes in lysosomal content of the cell, and alteration in lysosomal membrane permeability (Moore, 1988, 1990). These lysosomal responses, also called lysosomal activation, are related to enhanced autophagy following pollutant exposure and typically appear as increment in lysosomal size, and enhancement of the lysosomal/cell volume ratio with frequent swelling of the lysosomes and increases in hydrolase activities in mussels (Moore, 1988; Moore et al., 1996; Lowe and Fossato, 2000; Marigómez et al., 2005a,b).

The present work addresses (i) if there is any functional relation between CA activity in the digestive gland and the well-developed lysosomal compartment characteristic of digestive cells, and (ii) if CA activity is involved in the lysosomal system response to metal exposure.

To this end, the study utilized an *in vivo* exposure approach on *Mytilus galloprovincialis*. This filter feeding organism represents a choice model organism for investigating physiological responses to metals, because it is known to bioaccumulate trace metals in its tissues, and, in turn, to cope with high concentrations of these elements. Cadmium was used for exposure experiments. It is known to be one of the most toxic and world-wide diffused trace metal. It can be found in coastal and estuarine environments where it is mainly released by human activities such as smelting, mining, battery manufacturing, and pigment and plastic production (Pinot et al., 2000). Its toxic effects on aquatic organisms have been widely described (Lionetto et al., 1998b, 2000; Calisi et al., 2008).

## Methods

### Materials

Mussels, *Mytilus galloprovincialis*, were purchased from a local farm (Varano, Foggia, Italy). An homogeneous stock of mussels of the same size (shell length: 6.6 ± 0.5 cm; shell width: 3.5 ± 0.1 cm) was utilized. All experiments were performed in accordance with the Italian Animal Welfare legislation (D.L. 26/2014) that implemented the European Committee Council Directive (2010/63 EEC).

Unless otherwise indicated, all chemicals were purchased from Sigma Aldrich (St. Louis, MO, USA) and were of analytical grade.

### Experimental design

Four experiments were undertaken. Before each experiment, the animals, an individual per liter, were acclimated for 1 week in artificial seawater (Instant Ocean, Aquarium Systems, Inc., Wickliffe, Ohio) in controlled conditions of water temperature (15 ± 1°C), O_2_ concentration (saturating concentration), salinity (35 ± 1‰), and light:dark regime (12:12 h). After the acclimation, mussels were fed with 20 mg of a commercially available plankton preparation (CARMAR, Microplankton, San Giorgio a Cremano, Italy) added daily per liter.

Experiment n.1 was addressed to study the distribution of CA activity in several tissues of *Mytilus galloprovincialis* and to ascertain the intracellular localization of CA proteins expression in the digestive gland. Ten animals were used, the digestive gland, mantle and gills were sampled and the tissues were stored at −80°C for CA activity determination. Small fragments of digestive gland were removed, embedded in optimal cutting temperature (O.C.T) compound, frozen in liquid nitrogen and stored at −80°C for CA localization by immunofluorescence microscopy.

Experiment n.2 was carried out to ascertain *in vivo* the role of CA in lysosomal acidification in mussel digestive gland. Seventy-two specimens were utilized and a three factors experimental design was chosen: factor 1, “acetazolamide exposure” which included two levels (0 and 50 mg/l acetazolamide), factor 2, “time of exposure” with two levels (0 and 7 days), factor 3 “tank replication” which included two levels (two 10 L tanks for each condition with 9 animals in each tank replicate). Factor 1 and 2 were orthogonal and fixed, while factor 3 was nested in the combination 1 × 2. The animals were exposed in a semi-static regime. Water was changed every 48 h, and fresh acetazolamide added in the acetazolamide exposure tanks. Control animals were maintained in the same conditions of test organisms except for the absence of the drug. *M. galloprovincialis* CA in digestive gland was previously demonstrated to be highly sensitive to *in vitro* ACTZ exposure (Caricato et al., 2010). The ACTZ concentration used for the *in vivo* exposure experiment was one order magnitude lower that the concentration used by Zito et al. (2015) for 48 h exposure of embryos and larvae of other invertebrate species and corresponded to the concentration injected by Dietz (1978) in the freshwater mussel, *Carunculina texasensis*. After the exposure, mussels were dissected; the digestive gland was excised and immediately utilized for the cell suspension preparation and Lysosensor^TM^ Green staining. The factor “tank replicate” was not significant at *p* = 0.05 in any exposure experiments and this allowed the pooling of the nested component “tank replicate” in the ANOVA test. Therefore, data were analyzed by two-way analysis of variance (ANOVA).

Experiment n.3 was aimed to assess if the response of digestive gland CA to *in vivo* cadmium exposure was paralleled by the activation of the lysosomal system in digestive cells. One hundred and sixty-two mussels were utilized and a three factors experimental design was chosen: factor 1, “CdCl_2_ exposure” which included three levels (0, 100, and 300 μg/l CdCl_2_), factor 2 “time of exposure” which included three levels (0, 7, and 14 days), and factor 3 “tank replication” (see above). Factors 1 and 2 were fixed and orthogonal to each other, factor 3 was nested in the combination 1 × 2. The animals were exposed to cadmium in a semi-static regime for 7 or 14 days as reported above. Every 48 h fresh cadmium was added in the cadmium exposure tanks. Cadmium concentration in seawater was analyzed by atomic absorption spectrophotometry (Varian SpectrAA-880Z spectrometer) every 2 days in control and test tanks. During the exposure experiment cadmium concentrations remained closed to nominal concentrations (the percentage variation with respect to nominal concentration was lower than 10%).

The CdCl_2_ concentrations used were chosen for clearly addressing our working hypothesis in laboratory experimental conditions. They were lower than the *M. galloprovincialis* LC_50_ determined in larvae (0.59 mg/l) (Annicchiarico et al., 2007) and adults (1,6 mg/l) (Vlahogianni and Valavanidis, 2007) and about 3 ÷ 8 times higher with respect to environmentally realistic concentrations (Zhao et al., 2014). Moreover, 100 μg/l CdCl_2_ was already used in a previous study by Pytharopoulou et al. (2011) to detect the activation of an oxidative stress response in mussel exposed to Cd for 15 days without any reduction in the protein synthesis efficiency.

The mortality remained at low levels (<5%) during the exposure experiment. After the exposure, mussels were dissected and the digestive gland excised. One-half of each digestive gland was immediately utilized for cell suspension preparation, and the other half was stored at −80°C for CA activity determination. Data were analyzed by two-way analysis of variance (ANOVA) as reported above after the pooling of the nested component “tank replicate” in the ANOVA test.

Experiment n.4 was aimed to test the hypothesis of the involvement of CA in the response of the lysosomal system to *in vivo* cadmium exposure. A three factors experimental design was chosen: factor 1 “CdCl_2_ exposure” which included two levels (0 and 300 μg/l CdCl_2_), factor 2 “acetazolamide exposure” which included two levels (0 and 50 mg/l), and factor 3 “tank replication” (see above). Factors 1 and 2 were fixed and orthogonal to each other, factor 3 was nested in the combination 1 × 2. The exposure experiment was replicated at both 7 and 14 days. Overall two hundred and sixteen mussels were utilized (one hundred and eight for the exposure experiment at each time). Data were expressed as relative fluorescence, calculated as percentage of time 0 value and were analyzed by two-way analysis of variance (ANOVA) following arcsine transformation.

### CA activity determination

CA enzymatic activity was measured on tissue homogenate according to Caricato et al. (2010). Briefly, the homogenate was obtained by homogenizing the digestive gland in five volumes of 200 mM Mannitol, 0.2 mM HEPES–Tris, pH 8.5. The homogenization buffer contained PMSF (phenylmethylsulfonyl fluoride, 0.5 mM final concentration) and Leupeptin (0.006 mM final concentration) as antiproteolitic agents, and β-mercaptoethanol (0.01% final concentration) as a reducing agent. The reaction medium was composed as follows: 4 ml of Mannitol (200 mM), HEPES–Tris (0.2 mM) (pH 8.5 with KOH), 1.5 mg of tissue homogenate proteins, 6 ml of HEPES (3.5 mM) Tris (9.7 mM) (pH 8.5 with KOH) (0°C). The reaction was started at 0°C by the addition of 5 ml of CO_2_ saturated water and the pH recorded by a pH-meter (Seven Multi pH-Meter, Mettler Toledo, Greifensee, Switzerland). CA activity units were calculated from the initial rate of H^+^ production in the reaction mixture against a blank without the enzyme.

### CA localization by immunofluorescence confocal microscopy

Digestive gland cryosections (10 μm) were placed on poly-L-lysine coated glass, then fixed with paraformaldehyde (4% in PBS) for 10 min and washed three times with PBS. Sections were washed twice in saponin buffer, treated with blocking buffer for 1 h and then incubated for 1 h with the polyclonal antibody directed against CA II from human erythrocytes (Rabbit Anti-Carbonic Anhydrase II—Chemicon International, Temecula, CA, U.S.A.) (1:100), whose immunoreactivity against CA in mussel digestive was previously tested (Caricato et al., 2010). After washing, the sections were incubated with AlexaFluor 488 goat anti-rabbit IgG (1:1,000) as secondary antibody. Nucleus counterstaining was performed by propidium iodide treatment (5 μg/ml in citrated buffer), preceded by DNase-free RNAsi 100 μg/ml incubation (20 min).

Using a mounting medium containing the antifade agent 1,4-Diazabicyclo-octane (DABCO), and were observed under a confocal laser scanning microscope system (LSM 700 Zeiss, ZEN Software, GmbH, Germany). Laser beams with 488 and 555 excitation wavelengths were used to detect the green fluorescence from AlexaFluor 488, and the red fluorescence of propidium iodide utilizing a Plan-Apochromat 63 × /1.40 immersion-oil DIC objective.

### Preparation of digestive gland cell suspension

Preparation of digestive gland cell suspension was performed according to the method described by Faucet et al. (2004) with small modifications. Briefly, tissues were washed twice in calcium-magnesium free saline (CMFS) buffer pH 7.3 (20 mM HEPES, 500 mM NaCl, 12.5 mM KCl, 5 mM EDTA). Then, they were placed in a beaker containing CMFS buffer (20 ml per g of tissue) and minced into small pieces (2 mm) using scissors. The glands were stirred gently for 45 min with a magnetic stirrer (100 rpm). Cell suspensions were filtered on nylon filters through 100 μm and 50 μm-mesh respectively. The filtrate was centrifuged at 100 g for 10 min and pelleted cells were centrifuged again in CMFS buffer for 10 min. Cell viability was assessed by the *Trypan blue* exclusion test.

### Lysosensor™ green staining, spectrofluorimetric measurement, and confocal analysis

The cell pellet was resuspended at the final protein concentration of 0.6 mg/ml and incubated with 1 μM LysoSensor Green DND-189 (Molecular Probes Invitrogen Detection Technologies, Eugene, OR, USA) for 15 min in the dark. This is an acidotropic dye, which accumulates in acidic organelles as the result of protonation. The protonation also reduces the fluorescence quenching of the dye by its weak base side chain and this results in the pH-dependent increase in fluorescence intensity upon acidification (Haugland, 2005). With its low pK_a_ value, LysoSensor Green DND-189 is almost non-fluorescent except when inside acidic compartments. The cells were washed by centrifugation and resuspended in fresh CMSF buffer.

For spectrofluorimetric analysis, 100 μl of loaded cells (final protein concentration of the cell suspension: 0.2 mg/ml) were added to each well (final volume 300 μl with CMSF buffer) in a 96 well plate. Fluorescence intensity was recorded at λ_ex_ 443 nm and λ_em_ 505 using a spectrofluorometer reader (Synergy Mx, BioTek, Winooski, VT, U.S.A.). Fluorescence of unloaded cells, due to autofluorescence and light scattering, was subtracted from all spectrofluorimetric readings.

Preliminary *in vitro* assays allowed to assess that acetazolamide does not affect the LysoSensor Green DND-189 fluorescence.

For confocal microscopy analysis, 10 μl of loaded cells were immediately visualized with a LSM 700 (Zeiss, ZEN Software, GmbH, Oberkochen, Germany). Laser beam with 488 excitation wavelengths was used to detect the fluorescence of LysoSensor Green DND-189 utilizing a Plan-Apochromat 63 × /1.40 immersion-oil DIC objective.

The ratio of the fluorescent area with respect to the total cell area was performed by single cell analysis. The cell area and the lysosomal compartment area of suspended digestive gland cells were calculated by the ZEN software (Zaiss, Oberkochen, Germany). Approximately 80 cells per sample were analyzed.

### Statistical analysis

Data were analyzed by Analysis of Variance (ANOVA), using the WinGmav 5 software (designed, coded and complied by A.J. Underwood and M.G. Chapman, Institute of Marine Ecology, University of Sydney, Australia). The homogeneity of variance was tested by Cochran's test and the normality of data was assessed by Kolmogorov-Smirnov test prior to the application of ANOVA. Newman Keuls post-test was applied.

## Results

### Carbonic anhydrase in mussel digestive gland

**Figure 1:**
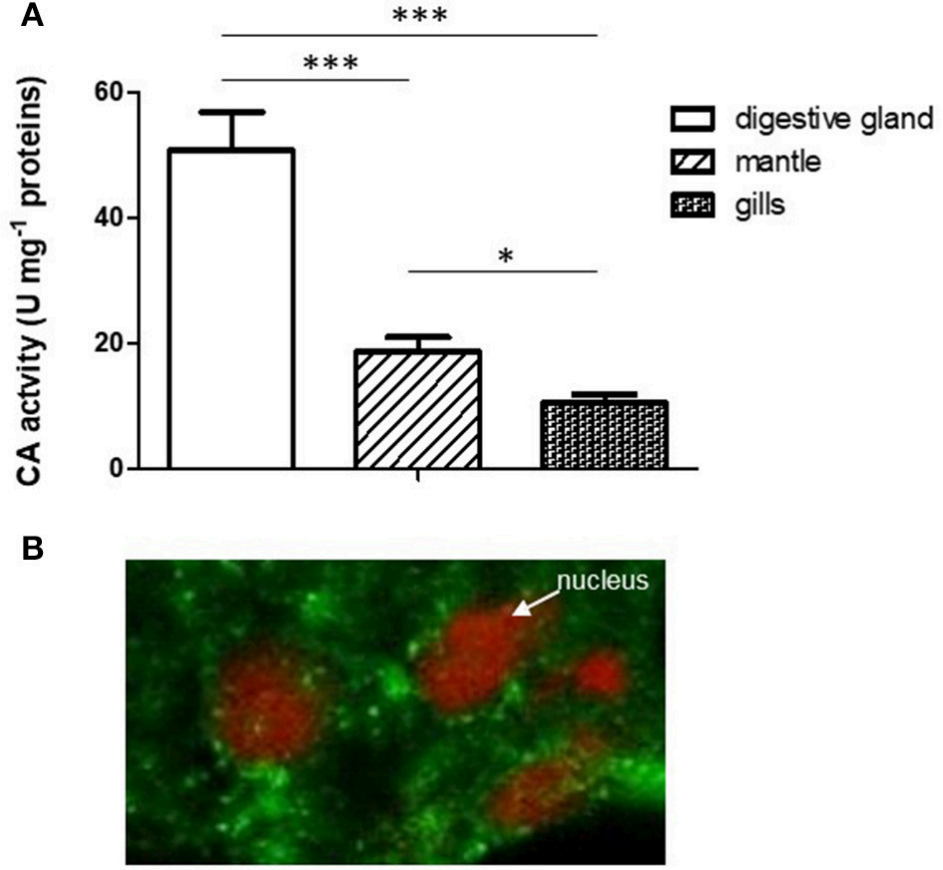
**(A)** Comparison of CA specific activity in digestive gland, mantle and gills. Data are expressed as mean ± SD (see Experiment n. 1 in the Methods section). The statistical significance of data was analyzed by One Way ANOVA. **(B)** Localization of CA in mussel digestive gland by confocal immunofluorescence analysis. The sections (10 μm) were incubated with the polyclonal rabbit Anti-Carbonic Anhydrase II and Alexa Fluor 488 goat anti-rabbit IgG (as secondary antibody) and visualized using confocal laser scanning microscopy as described (see Methods) utilizing a Plan-Apochromat 63 × /1.40 immersion-oil DIC objective. ^*^p < 0.05; ^***^p < 0.001.

A relevant specific CA activity was observed in mussel digestive gland, significantly higher with respect to the activity found in mantle and gills (Figure 1A). This result argues in favor of a relevant physiological role played by CA in this organ.

The localization of CA in the digestive gland was performed by confocal immunofluorescence microscopy on 10 μm tissue sections using a polyclonal antibody directed against CA II from human erythrocytes, previously tested on mussel digestive gland by Western blot analysis (Caricato et al., 2010). As reported in Figure 1B, the enzyme showed a cytoplasmic punctate localization in digestive cells double marked for nuclei staining with propidium iodide (red) and CA immunodetection (green).

### Acetazolamide effect on lysosomal acidification

In order to demonstrate *in vivo* the direct involvement of CA in lysosomal acidification, specimens of *M. galloprovincialis* were exposed to 50 mg/l acetazolamide, a specific CA inhibitor, for 7 days (second experiment, see Methods). Then, the digestive cells from control and treated animals were isolated and charged with the fluorescent acidotropic dye LysoSensor Green DND-189 that exhibits a pH-dependent increase in fluorescence intensity upon acidification.

The acetazolamide concentration utilized for the *in vivo* exposure experiments represents a sub lethal concentration (Zito et al., 2015). The mortality value observed in the acetazolamide treated group was statistically indistinguishable from the mortality observed in the control group and was lower than 5%.

**Figure 2:**
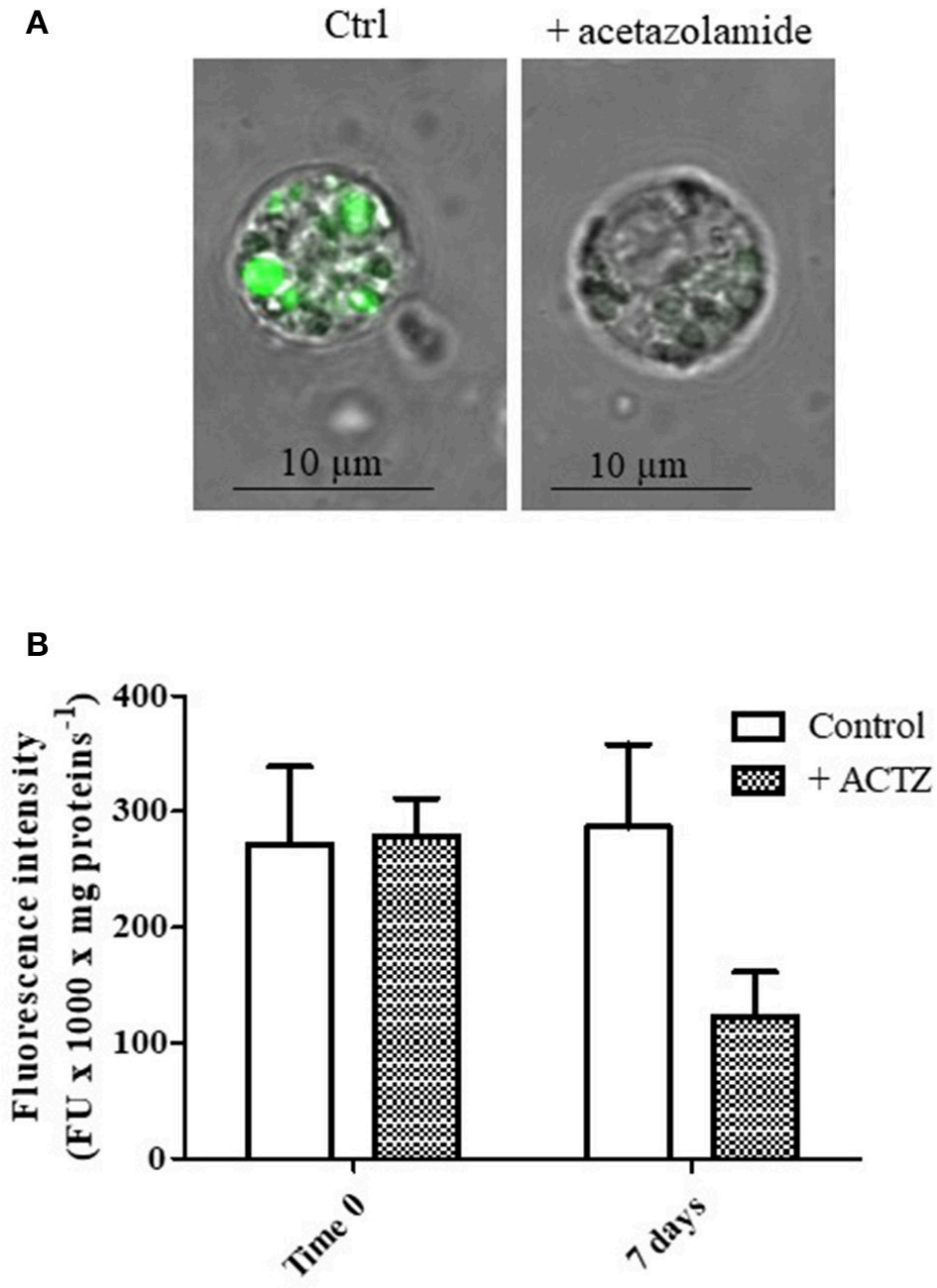
**(A)** Appearance of digestive cells stained with LysoSensor Green DND-189 and observed by confocal laser scanning microscopy utilizing a Plan-Apochromat 63 × /1.40 immersion-oil DIC objective. Cell were obtained from control and acetazolamide exposed (7 days) mussels (see Experiment n.2 in the Methods section). **(B)** Fluorescence intensity of digestive cells in suspension from control and acetazolamide exposed (7 days) mussels stained with LysoSensor Green DND-189 and analyzed by a spectroflurimeter (see section Methods). The fluorescence intensity measurements (expressed as fluorescence units) were normalized to the protein content of each sample. Data are expressed as mean ± SD. The statistical significance of data was analyzed by two way ANOVA and Newman-Keuls post-test.

Figures 2A,B show the representative appearance of isolated digestive cells marked with LysoSensor Green DND-189 of control and acetazolamide treated animals. As observed in the control digestive cells (Figure 2A), the probe florescence was confined in vesicular structures. The different intensity in the green color of the vesicles could correspond to different intravesicular pH values. Vesicles marked in brilliant green could correspond to secondary lysosomes, characterized by a lower pH, which can reach 2–5 μm diameter in digestive cells in physiological conditions (Moore et al., 1987). On the other hand acetazolamide exposure was able to markedly decrease the green fluorescence of the dye localized in the vesicles (Figure 2A).

The observation by confocal microscopy on single cell was paralleled by spectrofluorimetric analysis on cell suspensions (Figure 2B). The acetazolamide treatment was able to significantly inhibit the LysoSensor green fluorescence by about 58% (*p* < 0.01), demonstrating *in vivo* the involvement of CA in lysosomal acidification and, in turn, in lysosomal function.

### Response of CA and lysosomal system to cadmium exposure

**Figure 3:**
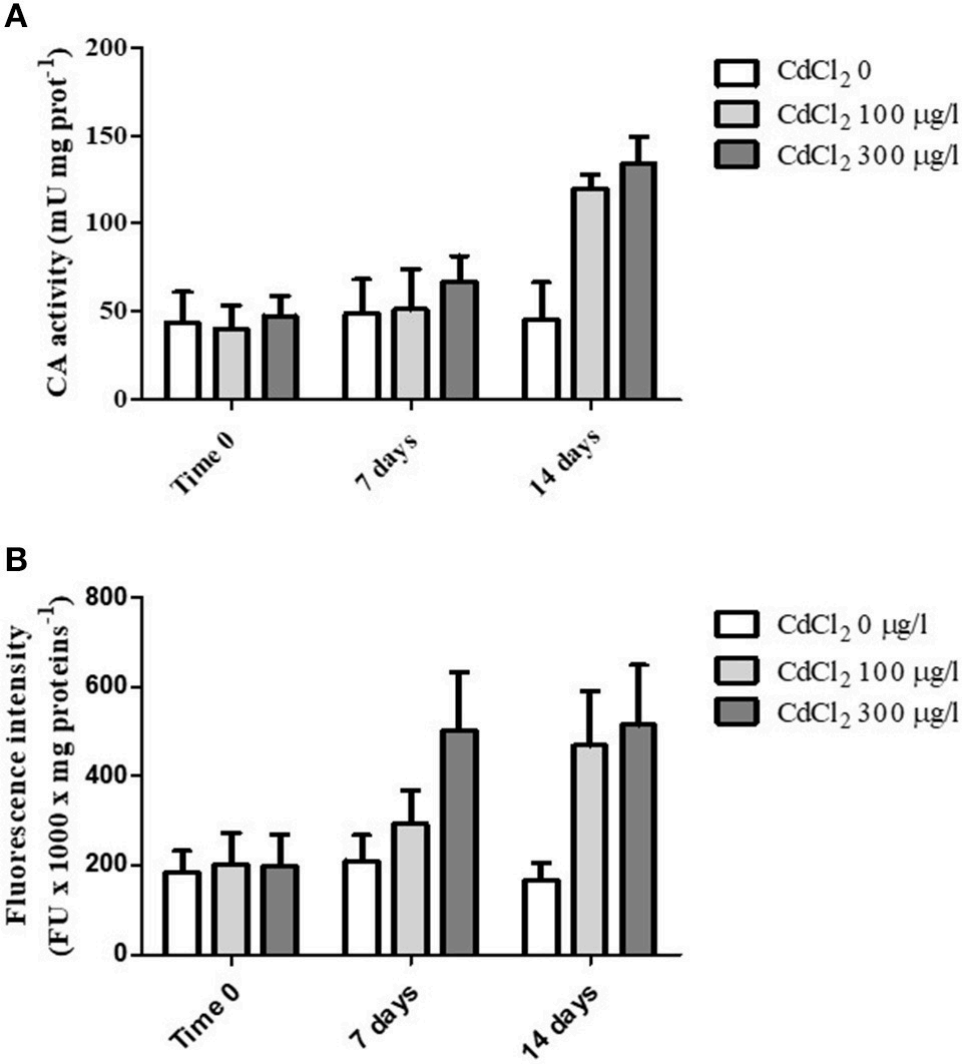
**(A)** CA specific activity measured in the digestive glands obtained from control and CdCl_2_ exposed mussels at time 0, 7 and 14 days (see Experiment n.3 in the Methods section). **(B)** Fluorescence intensity (expressed as fluorescence units/mg of proteins) of suspended digestive gland cells marked with LysoSensor Green DND-189 obtained from control and CdCl_2_ exposed mussels at time 0, 7 and 14 days (see Experiment n.3 in the Methods section). Data are expressed as mean ± SD. The statistical significance of data was analyzed by two way ANOVA and Newman-Keuls post-test.

When the animals were exposed for 2 weeks to 100 and 300 μg/l CdCl_2_ respectively, we observed a marked increase of the CA activity following 14 days of exposure at both concentrations tested (Figure 3A). The statistical analysis of data by two-way ANOVA showed that cadmium exposure had a highly significant (*p* < 0.001) effect on CA activity. Exposure time also had a significant (*p* < 0.001) effect, and the interaction between these two factors was in turn significant (*p* < 0.01), suggesting that cadmium exposure had an increasing effect on CA activity with the time.

Moreover, in parallel to CA activity determination we also analyzed the fluorescence intensity of digestive cells charged with LysoSensor Green DND-189 on the same specimens. The cells showed a marked increase in the green fluorescence at the two CdCl_2_ concentrations tested with time (Figure 3B). The increase was evident already after 7 days of exposure at 300 μg/l CdCl_2_ and reached its maximal value after 14 days for both concentrations. The statistical analysis of data revealed a significant effect of the two variability factors “cadmium exposure” and “time of exposure” (*p* < 0.001). The interaction between them was also significant (*p* < 0.01).

**Figure 4:**
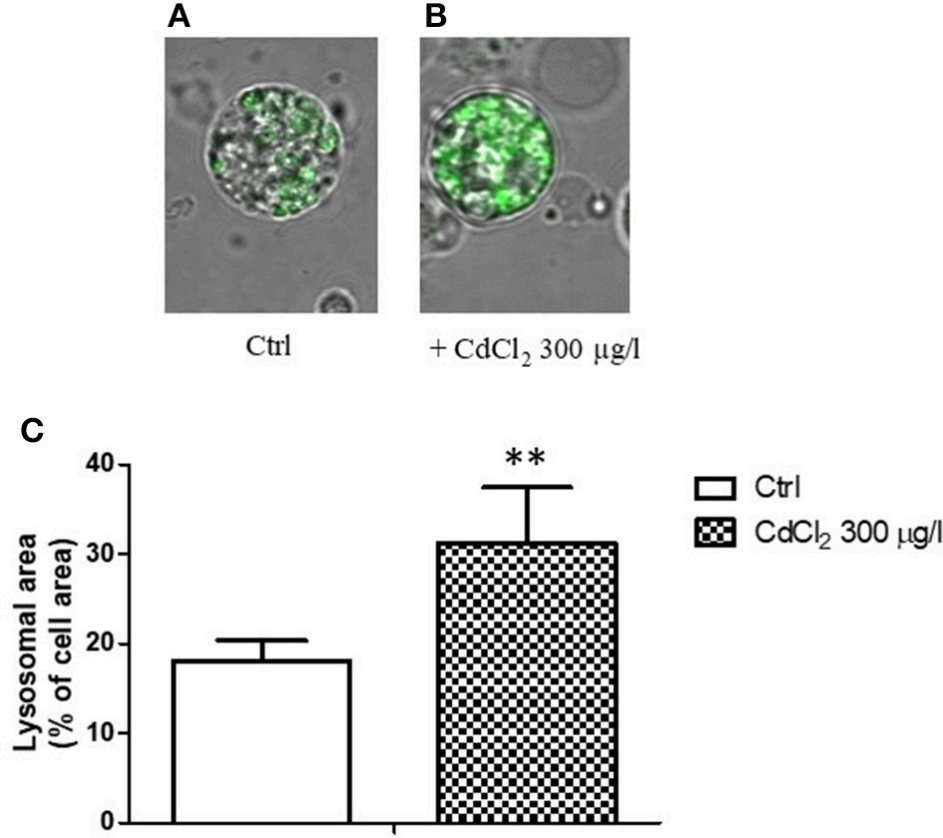
**(A,B)** Appearance of digestive gland cells from control **(A)** and 300 μg/l CdCl_2_ **(B)** exposed mussels (14 days) stained with LysoSensor Green DND-189 and observed by confocal laser scanning microscopy utilizing a Plan-Apochromat 63 × /1.40 immersion-oil DIC objective. **(C)** Lysosomal/cell area ratio determined on digestive gland cells from control and 300 μg/l CdCl_2_ exposed mussels (14 days) stained with LysoSensor Green DND-189 and observed by confocal laser scanning microscopy. Data are expressed as mean ± SD ^**^p < 0.01 (Student *t*-test).

When a single cell analysis was performed by confocal microscopy on isolated digestive cells, a significant increased ratio of the fluorescent area with respect to the total cell area was observed in cadmium exposed animals (Figures 4A–C), suggesting a proliferation and/or increase in size of lysosomes in cadmium exposure condition.

### Acetazolamide effect on the lysosomal response to cadmium exposure

**Figure 5:**
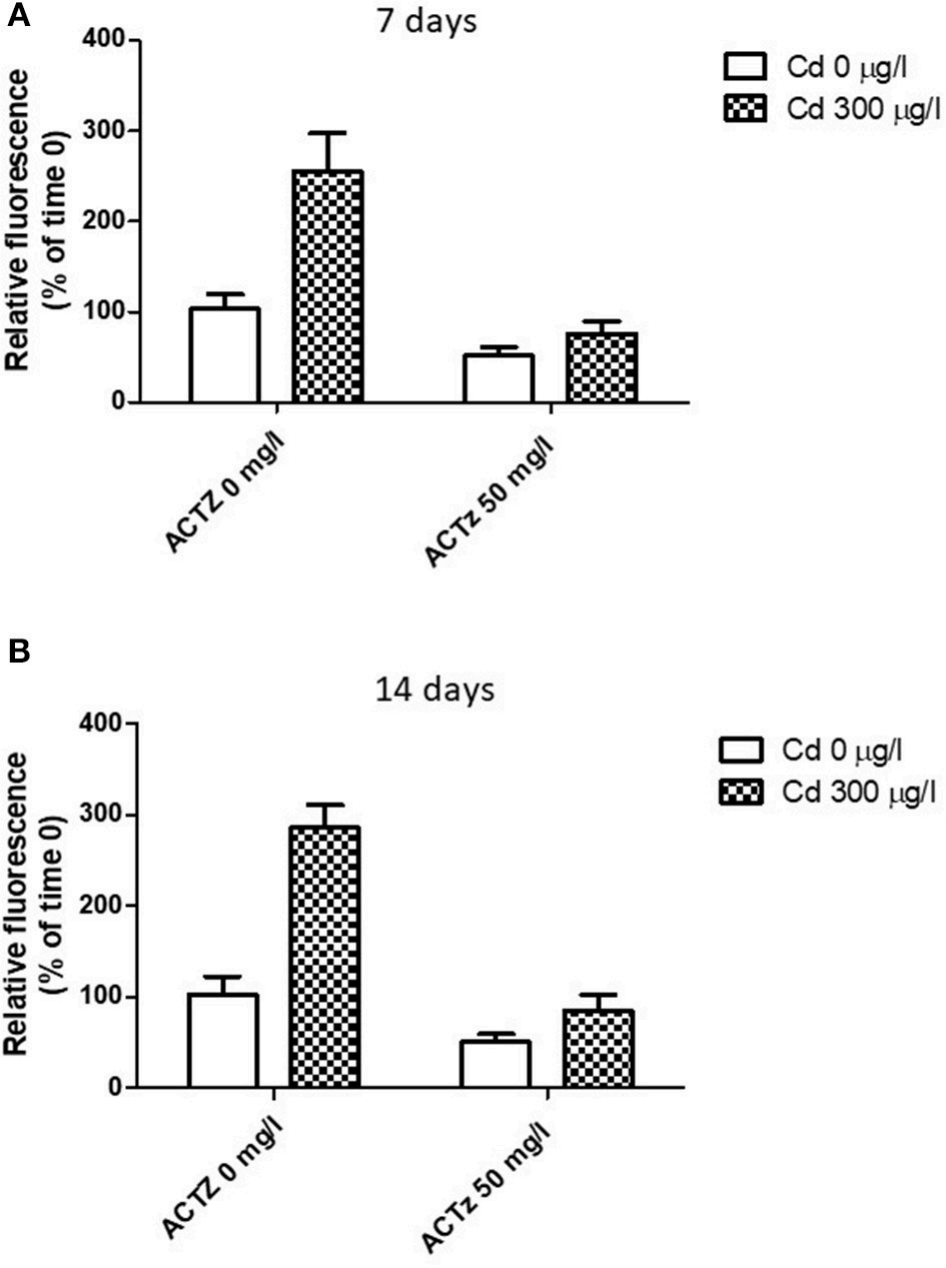
**(A,B)** Effect of acetazolamide and 300 μg/l CdCl_2_ exposure on the fluorescence intensity of digestive gland cells in suspension stained with LysoSensor Green DND-189 (see Experiment n.4 in the Methods section) following 7 and 14 days of exposure. Data are reported as the mean ± SD. Data are expressed as relative fluorescence calculated as percentage of time 0 value.

After having demonstrated a correspondence between CA increased activity and lysosomal response to cadmium exposure, we co-exposed the animals to cadmium (300 μg/l) and acetazolamide (50 μg/l) in order to assess if there was any interaction between the two events. The co-exposure was repeated for 7 and 14 days respectively (see Methods). Figures 5A,B shows the effect of acetazolamide on the CdCl_2_ induced LysoSensor Green fluorescence (expressed as relative fluorescence vs. time 0) in digestive gland cells in suspension following 7 and 14 days respectively. The interaction between the two variability factors was significant at each time (time 7 days: *p* < 0.05; time 14 days: *p* < 0.01). This means that in both cases, acetazolamide did not exert the same effect in control and CdCl_2_ 300 μg/l conditions. In fact, it exerted its partial inhibition (about 50%) on control organisms as reported above, but in cadmium exposed organisms its effect was significantly higher. In fact, in this case acetazolamide was able to almost completely inhibit the metal induced increase of LysoSensor fluorescence and no significant difference was observed between Cd 0 μg/l and CdCl_2_ 300 μg/l in the presence of the drug.

## Discussion

In the present work, we investigated the functional role of CA in the digestive gland of *Mytilus galloprovincialis* and its possible involvement in the lysosomal response to heavy metal exposure by using an *in vivo* approach.

The digestive gland showed a significantly higher specific CA activity with respect to other organs such as gills and mantle, where CA has been functionally related to CO_2_ exchange with the environmental water or calcium carbonate deposition in the shell (Medakovic, 2000; Cudennec et al., 2006; Le Roy et al., 2012; Werner et al., 2013; Pavičić-Hamer et al., 2016). This result argues in favor of a relevant physiological role played by CA in the digestive gland. This organ represents the key metabolic center in mollusks, being involved in energy storage, metabolic transformation, and trace element detoxification. Its cells are known to operate a fine regulation of intracellular pH (pHi), which is of crucial importance in cellular metabolism, by the activity of specific membrane transport systems, such as a Na^+^/H^+^ exchanger, a Cl^−^/${\text{HCO}}_{3}^{-}$ exchanger and a H^+^ pump (Manzl et al., 2004). CA is known to play a vital role in cellular acid/base balance, being a key element for the functioning of acid/base-coupled membrane transporter. Therefore, it is reasonable to hypothize that in the digestive gland CA contributes to the regulation of intracellular pH, which is of pivotal importance for metabolic processes.

Moreover, the exploration of the CA physiological contribution to the digestive gland functioning cannot ignore that digestive cells, the most abundant cell type comprising the epithelium of the digestive diverticula, are characterized by a very developed endo-lysosomal system (Moore and Simpson, 1992). This system is primarily involved in the uptake and digestion of food materials and then in pollutant accumulation and detoxification (Cajaraville et al., 1995; Marigómez et al., 1996; Domouhtsidou and Dimitriadis, 2001). CA, catalyzing the H^+^ production from metabolic CO_2_, could provide the H^+^ necessary for the lysosomal acidification by the operation of the lysosomal V-ATPase.

When the animals were exposed to acetazolamide (Experiment 2, see experimental plan), specific CA inhibitor, for 7 days, the lysosomal acidification was significantly inhibited (by about 58%), as assessed on LysoSensor green marked cell by confocal microscopy and spectrofluorimetric analysis. This result clearly demonstrated *in vivo* the physiological contribution of CA to the acidification of the lysosomal compartment. However, considering the partial effect of acetazolamide (about 58%), it is possible to hypothesize also the contribution of other sources, different from metabolic CO_2_, to lysosomal acidification, such as metabolic lactate production (Brisson et al., 2016).

The CA immunoreactivity analyzed in digestive gland by confocal microscopy showed a cytoplasmic punctate localization. Although this result needs further deepening, a possible association of CA to lysosomes cannot be excluded, considering that CA was found as a constituent of lysosomes in other cell types (PMNs, hepatocytes, tooth cells) (Rikihisa, 1985; Reibring et al., 2014). Future works will clarify this aspect.

When the mussels were exposed to cadmium 100 and 300 μg/l for 2 weeks, CA activity significantly increased. The increase was particularly evident following 2 weeks of exposure at both concentrations tested. This observation is in accordance with previous results showing a significant enhancement in the CA activity due to increased CA protein expression following to 1.785 μM CdCl_2_ exposure for 2 weeks (Caricato et al., 2010). In parallel, the LysoSensor green fluorescence of isolated digestive cells in suspension revealed an increment during the exposure to the metal. At 300 μg/l CdCl_2_, the fluorescence intensity was significantly increased at its maximal value already after 1 week exposure, while at 100 μg/l the increase was maximal following 2 weeks exposure. The single cell confocal analysis on isolated digestive cells from control and 300 μg/l cadmium exposed animals indicated an increase in the lysosomal area/cell area ratio suggesting a proliferation and/or increase in size of lysosomes in cadmium exposure condition. Increased lysosomal size has been previously found in response to chemical pollution and other sources of environmental stress by several authors (Cajaraville et al., 1995; Marigómez et al., 1996; Domouhtsidou and Dimitriadis, 2001; Marigómez and Baybay-Villacorta, 2003). In particular, Marigómez et al. (2005a) previously reported increased numbers of lysosomes and lysosomal enlargement in *M. galloprovincialis* digestive gland following cadmium exposure.

Timely comparing the CA activity and the Lysosensor fluorescence intensity in suspended cells in the exposed organisms, at both CdCl_2_ concentrations we observed a parallel increase in both variables following 2 weeks of exposure, while at 300 μg/l CdCl_2_ we observed an early increase only in the LysoSensor fluorescence.

From these results, it is possible to argue that under CdCl_2_ exposure CA induction could be functional to the prolonged increased requirement of H^+^ for supporting lysosomal acidification during lysosomal activation. It is possible that in a short term (7 days) the cell can sustain the increased H^+^ requirement at higher CdCl_2_ concentrations by the basal CA activity and/or the contribution of other acid sources. However, the induction of the enzyme could be functional to prolonged responses.

This result arises the intriguing question about the mechanisms underlying the regulation of CA expression. In plants and microalgae the CA *de novo* synthesis of CA is known to be influenced by some environmental factors, including trace metals (Lane and Morel, 2000; Khan et al., 2008). In animals, less information is available. Some works indicate CA protein expression to be influenced by Zn^2+^ availability, as it has been reported for CAVI in humans (Henkin et al., 1999), or for CAII in rats (Goto et al., 2008). In the fish *Cyprinodon variegatus*, CAII gene expression in gills and intestine was influenced by Cu^2+^ exposure in a concentration-dependent manner (De Polo et al., 2014). Our data suggest a metal inducibility of CA in mussel digestive gland, that needs to be deepened in a future work. If CA induction is related in a certain way to the gene network controlling biogenesis and regulation of lysosomes is another intriguing hypothesis that will be addressed in future studies.

Acetazolamide was able to completely inhibit the cadmium-induced increase of the LysoSensor fluorescence in isolated digestive cells exposed to 300 μM cadmium at both 7 and 14 days. This suggests that the cadmium induced LysoSensor fluorescence increase is dependent on CA activity as H^+^ supplier and in turn on metabolic CO_2_ during the time course of the exposure.

In conclusion, our results demonstrated the functional role of CA in the lysosomal acidification of digestive gland in *Mytilus galloprovincialis* and pointed out its involvement in the lysosomal activation following cadmium exposure through an *in vivo* approach. This work opens new perspective in the comprehension of the functioning of this enzyme in animals and in its regulation under the influence of environmental factors, such as trace metals.

## Author contributions

Conceptualization: ML and RC; methodology: RC, MG; investigation: RC, MG, TS, ML writing, original draft: ML and RC; writing, review and editing: ML and RC; funding acquisition: TS; Supervision: ML.

### Conflict of interest statement

The authors declare that the research was conducted in the absence of any commercial or financial relationships that could be construed as a potential conflict of interest.
